# Four Arguments against the Adult-Rating of Movies with Smoking Scenes

**DOI:** 10.1371/journal.pmed.1001078

**Published:** 2011-08-23

**Authors:** Simon Chapman, Matthew C. Farrelly

**Affiliations:** 1 University of Sydney, Sydney, Australia; 2 RTI International, North Carolina, United States of America

## Abstract

Simon Chapman and Matthew Farrelly argue against recent calls in the US and elsewhere for movies with smoking scenes to be adult-rated.

**Summary Points**

- In the US, a growing number of medical and public health agencies are calling for movies with smoking scenes to be adult rated. We present four arguments against such proposals.
- First, studies purporting to demonstrate causal associations between exposure to smoking in movies and smoking uptake do not control for large-scale confounding of the independent variable (smoking in movies).
- Second, claims for attributable uptake of smoking said to be caused by movie smoking exposure are crudely reductionist, ignoring widespread exposure to smoking scenes elsewhere.
- Third, adult classification is a highly inefficient way of preventing youth exposure to adult-rated content.
- Fourth, we have concerns about the assumption that advocates for any cause should feel it reasonable that the state should regulate cultural products like movies, books, art, and theatre in the service of their issue.

A few years ago the Indian government tried unsuccessfully to ban all smoking scenes in movies [1]. Thailand pixelates cigarettes on television [2]. In the US, over 20 public health agencies and the World Health Organization [3] are campaigning to require that most smoking scenes should trigger restricted (adult) film classification [4]. They believe that such ratings would significantly reduce the exposure of youth to smoking scenes in movies, which they argue directly cause the uptake of smoking [5].

Should this policy become adopted, historical figures (such as King George VI in the 2011 Oscar winning *The King's Speech*) will still be able to smoke in films rated as acceptable for children. But nameless or fictional smokers would cause an adult rating, regardless of the historical or cultural accuracy of such casting, unless they were scripted to openly proselytize against smoking. Apparently it is unreasonable to airbrush the historical record of a well-known individual's smoking, but defensible for this to occur where whole populations or eras are concerned.

Efforts should be applauded to expose and outlaw paid tobacco industry product placement in film [6],[7]—which is unarguably a form of advertising—as well as efforts to raise awareness within the film and television industries about the ways that gratuitous depiction of smoking can assist in normalizing smoking. However, we have four concerns about the ratings classification proposal: two methodological, one practical, and one a matter of principle.

The first is the major problem in the evidence base of movie smoking scenes being inextricably entangled with a host of other variables in movies. The research bedrock of the restricted ratings proposal is a growing body of research said to satisfy criteria that exposure to smoking in movies causes smoking in youth [5], including that there is a dose-response relationship between movie smoking exposure and likelihood of smoking uptake [8]. While the best of these studies control for several of potentially many subtle confounding factors associated with the dependent variable (youth who do and don't smoke) such as social, parental, and youth psychological factors like self-assessed “rebelliousness” and risk-taking, clearly this can be only half the story. Some might also argue that with rebelliousness being measured by items like “I like to do scary things” and “I like to listen to loud music” [9], that the scales used to measure such constructs may be rather dated and of dubious validity. But, critically, potential important covariates of the independent variable (smoking in movies) are never considered. Smokers in movies never just smoke. And movies showing smoking have a lot more in them that might appeal to youth at risk of smoking than just smoking. Why is this “muddying” of the independent variable a critical consideration? Let us explain.

Teenagers select movies because of a wide range of anticipated attractions gleaned from friends, trailers, and publicity about the cast, genre (action, sci-fi, teen romance, teen gross-out/black humour, survival, sports, super hero, fantasy, and so on), action sequences, special effects, and soundtrack. It is likely that youth at risk for current or future smoking self-select to watch certain kinds of movies. These movies may well contain more scenes of smoking than the genres of movies they avoid (say, parental-approved “family friendly,” wholesome fare like the *Narnia Chronicles* or *Shrek*).

Teenagers at risk of smoking are also at higher risk for other risky behaviors [10] and comorbidities [11]. They thus are likely to be attracted to movies promising content that would concern their parents: rebelliousness, drinking, sexual activity, or petty crime. Smoking will often be part of such movie tableaux, along with many other hard-to-quantify variables (character “attitude,” irreverence, fashion sense) where the subtle and ever-changing semiotics involved present significant problems for questionnaire-based data gathering required for the calculation of attributable risk estimates (see below). Movie selection by those at risk of smoking is thus highly relevant to understanding what it might be that characterizes the association between young smokers having seen many such movies and their subsequent smoking. Movie smoking may be largely artifactual to the wider attraction that those at risk of smoking have to certain genres of films. These studies rarely consider this rather obvious possibility, being preoccupied with counting smoking in the films.

By assuming that seeing smoking in movies is causal, rather than simply a marker of movie preferences that have more smoking in them than the movie preferences of those less at risk, authors fail to consider problems of specificity in the independent variable (movies with “smoking”). It may be just as valid to argue that preferences for certain kinds of movies are predictive of smoking. The putative “dose response” relationships reported may be nothing more than reporting that youth who go on to smoke are those who see a lot of movies where smoking occurs, among many other unaccounted things.

If researchers also coded for potential covariates such as alcohol or recreational drug portrayal, violence, coarse language, and sexual content, the depth of these very muddied waters might become apparent. One rare US study to have done this examined 71 top-grossing films over a 4-year period and found that “the correlation between exposure to smoking in the movies and other adult content (nudity, violence, profanity) was so high [0.995] that it was impossible to disentangle their separate influence.” [12]. A challenge to the field would be to identify a subset of movies with much smoking, but no confounders like profanity, nudity, and violence, to examine variation in exposure to such movies and any relationship with subsequent smoking.

Our second concern is with the crude reductionism and questionable precision evident in the reasoning that allows conclusions like “390,000 [US] kids [are] recruited to smoke each year by the smoking they see on screen” [13], that introducing adult rating would prevent “probably 200,000 a year from starting to smoke” [14], and that smoking in movies claims “120,000 lives a year” [14] in just the US. This epidemiological alchemy invites us to accept that these legions of children only smoke because of their exposure to movie smoking and that the resilience of this influence is so great that it retains a vice-like grip all the way through to the eventual death of these young smokers decades later, unmodified by other influences throughout these years. A lifetime of exposure to the sight of smoking in uncounted public, social, and family situations; years of exposure to tobacco advertising and promotions still rampant in the US and many other nations; exposure to smoking scenes often by the same influential movie and music stars in magazines [15], music videos [16], and on YouTube [17]—indeed “all the above” and more are ignored because of the impossibility of reliably quantifying such ubiquitous exposure over many years.

If the adult classification system for smoking was adopted, it would seem likely that the same youth at risk for smoking would still go to the same (then smoking-expurgated) kinds of films they now prefer: they don't select them only in anticipation of seeing smoking, the sight of which is commonplace. Meanwhile, they would still see copious amounts of on-screen smoking in the adult-rated films they already see with consummate ease as well as all the other daily sightings of smoking that are conveniently not considered in these sorts of studies.

This leads to our third concern: the naivety of policy advocacy that assumes that film classification actually prevents young people from seeing “forbidden fruit.” Glantz—a leading advocate for adult classification—has pilloried efforts to stop shopkeepers selling tobacco to minors because youth are street-smart enough to get older friends to buy cigarettes [18]. But similarly, youth very frequently access adult-rated movies via friends and download them legally and illegally by the millions from the web. In the US in 2008, an estimated 10.397 million children aged 12–17 watched a movie on the internet, 5.6 times more than those who downloaded music. The average US teen saw 31.4 movies, of which only 10.8 (34%) were seen at a cinema [19]. Nearly all (98.9%) 15-year-old Swedish boys and 73.5% of girls have viewed pornography, often accessed through file-sharing sites [20]. Beliefs that restricting cinema viewing of smoking to adults is a workable solution seem rapidly irrelevant with the exponential changes brought by the Internet.

Fourth, and most fundamentally, we are concerned about the assumption that advocates for any cause should feel it reasonable that the state should regulate cultural products like movies, books, art, and theatre in the service of their issue. We believe that many citizens and politicians who would otherwise give unequivocal support to important tobacco control policies would not wish to be associated with efforts to effectively censor movies other than to prevent commercial product placement by the tobacco industry.

The role of film in open societies involves far more than being simply a means to mass communicate healthy role models. Many movies depict social problems and people behaving badly and smoking in movies mirrors the prevalence of smoking in populations [21]. Except in authoritarian nations with state-controlled media, the role of cinema and literature is not only to promote overtly prosocial or health “oughts” but to have people also reflect on what “is” in society. This includes many disturbing, antisocial, dangerous, and unhealthy realities and possibilities. Filmmakers often depict highly socially undesirable activities such as racial hatred, injustice and vilification, violence and crime. It would be ridiculously simplistic to assume that by showing something most would regard as undesirable, a filmmaker's purpose was always to endorse such activity. Children's moral development and health decision-making occurs in ways far more complex than being fed a continuous diet of wholesome role models. Many would deeply resent a view of movies that assumed they were nothing more than the equivalent of religious or moral instruction, to be controlled by those inhabiting the same values.

The reductio ad absurdum of arguments to prevent children ever seeing smoking in movies would be to stop children seeing smoking anywhere.

The call for movies with smoking to be adult rated has been almost wholly conducted within the US, where some 70% of Americans agree that smoking scenes should cause a movie to be thus rated [22]. Many Americans also believe in devil possession (58.6%), a biblical rather than evolutionary account of the origins of life (55.8%), UFOs (40.6%), and astrology (33.3%) [23]. The popularity of beliefs is not always a reliable guide to their wisdom. Such reactions perplex many outside the US who have long been used to far more relaxed regulation of film and television.

Proponents of the rating system for smoking argue that their proposal simply seeks to extend to smoking scenes the ratings system that now operates for sex and violence. Adult-rating advocates like to argue that smoking in movies should be treated identically to coarse language. However, non–adult-rated movies in many other nations frequently contain swearing, moderate violence, and sex scenes where panels appointed to judge the rating for the entire film have decided that these scenes do not overwhelm the overall suitability of the film to be screened to children. These panels are typically not constrained by prescribed formulae as would appear to be the case with swearing in the US, but asked to make a holistic judgment with reference to unspecified community standards.

The US has First Amendment constitutional problems in banning above-the-line tobacco advertising [24] and largely because of this remains one of the few nations to have still not ratified the WHO's Framework Convention on Tobacco Control, which requires all tobacco advertising to be banned. Its public health community may therefore be drawn to advocacy for controls that they feel have some hope of progressing domestically such as film classification. But other than in India and Thailand, we are aware of no significant momentum in governments or tobacco control circles for this to occur. This nascent momentum toward censorship and classification of smoking in movies deserves critical scrutiny from all who cherish open, civil society.
